# Mapping and Functional Characterization of *Stigma Exposed 1*, a *DUF1005* Gene Controlling Petal and Stigma Cells in Mungbean (*Vigna radiata*)

**DOI:** 10.3389/fpls.2020.575922

**Published:** 2020-11-19

**Authors:** Yun Lin, Kularb Laosatit, Jingbin Chen, Xingxing Yuan, Ranran Wu, Kitiya Amkul, Xin Chen, Prakit Somta

**Affiliations:** ^1^Institute of Industrial Crops, Jiangsu Academy of Agricultural Sciences, Nanjing, China; ^2^Department of Agronomy, Faculty of Agriculture at Kamphaeng Saen, Kasetsart University, Nakhon Pathom, Thailand; ^3^Center of Excellence on Agricultural Biotechnology: (AG-BIO/PERDO-CHE), Bangkok, Thailand

**Keywords:** exposed stigma, floral structure, cell morphology, gene cloning, mungbean

## Abstract

Flowers with exposed stigma increase the outcrossing rate and are useful in developing improved hybrid crop cultivars. This exposure results mainly from the cellular morphology of the petal and pistil, but what affects the formation of the petal and pistil in the late developmental stages is less understood. Here, we characterized a novel floral mutant in mungbean (*Vigna radiata*), *stigma exposed 1* (*se1*), which displays irregular petals and pistils. Floral organ initiation in the *se1* mutant was normal, but petal and pistil growth malfunctioned during late development. A histological analysis revealed that the *se1* mutant had wrinkled petals with knotted structures and elongated styles. The cellular morphology of the epidermal layers of the *se1* petals was deformed, while the cell lengths in the styles increased. A genetic analysis indicated that the *se1* phenotype is controlled by a single recessive gene, and it was mapped to chromosome 11. A sequence analysis suggested that a DUF1005-encoding gene, *LOC106777793*, is the gene controlling the *se1* phenotype. The *se1* mutant possessed a single-nucleotide polymorphism that resulted in an amino acid change in VrDUF1005. Overexpression of *VrDUF1005* in *Arabidopsis* resulted in rolling leaves and reduced floral size. Consequently, we proposed that *VrSE1* functions to modulate cell division in petals and cell expansion in styles during the late developmental stages in mungbean. The *se1* mutant is a new genetic resource for mung bean hybrid breeding.

## Introduction

Owing to a growing global population, the world is facing a food security problem, and hybrid breeding, utilizing heterosis, is an effective way to improve crop yields (Schnable and Springer, 2013). The efficiency of pollen grain transfer to stigmas through pollinators (e.g., insects or wind) determines the commercialization of the hybrid seeds; however, hybrid breeding for autogamous plants has had limited success owing to the inefficiency and time-consuming nature of conventional manual emasculation (Longin et al., 2012). Most legume crops are predominantly self-pollinating. To date, pigeon pea is the only successful example of legume commercial hybrid breeding (Saxena et al., 2013), which was possible through the development of a cytoplasmic nuclear male sterility (CMS) line through an interspecies cross (Saxena et al., 2005). As a *Vigna* genus (Family Leguminosae) member, mungbean (*Vigna radiata* L.) has a typical papilionaceous flower structure with one standard, two wings, and two keel petals, although some leguminous species possess complex floral morphologies to adapt to insect pollinators (Westerkamp, 1997; Aronne et al., 2012; Guo et al., 2019). The anthers and one stigma of mungbean are enclosed in the “keel–wing” petal complex before fertilization; such mating behavior results in an extremely low outcrossing rate, approximately only 1.7% for mungbean (Sangiri et al., 2007). Thus, the floral morphology is the major obstacle in mungbean commercial hybrid breeding. Stigmas that project above the anthers (i.e., approach herkogamy) significantly increase the outcrossing rate (Barrett et al., 2000; Barrett, 2003; Cronk and Ojeda, 2008). For example, exserted stigmas that extend pass the glume (homologous to petal) increase outcrossing rate in rice (Kato and Namai, 1987).

Our understanding of floral development has greatly increased in recent years. The classical “ABC” model has been widely proven to regulate floral organ identity (Coen and Meyerowitz, 1991). This model posits that A-class genes specify sepals, A- and B-class genes specify petals, B- and C-class genes act together to regulate stamen development, while C-class genes direct carpel identity, and A and C act in an antagonistic manner. However, the genetic mechanisms of late floral organ development such as petal shape and stigma length (stigma exertion) are less understood. Previous studies revealed that stigma exertion is a complex quantitative trait, and its expression is affected by style length, petal size, petal shape, and environments (Yan et al., 2009; Hermann and Kuhlemeier, 2011; Pan et al., 2019). In rice, several loci associated with stigma exsertion ratio have been identified by quantitative trait loci mapping or genome-wide association study (Uga et al., 2003a, b; Miyata et al., 2007; Takano-Kai et al., 2011; Zhou et al., 2017; Liu et al., 2019). Among those loci, *GS3* (*Grain Size3*) increases the cell number in the style, while *GW5* (*Grain Weight5*) alters glume size (Zhou et al., 2017). In *Solanum* plants, style polymorphism is a common phenomenon. A major QTL, *Se2*.*1*, modulating style length was mapped in an interspecific crossed population (Chen and Tanksley, 2004). A transcription factor, *LO2*, is the gene underlying the QTL *Se2*.*1*, which promotes cell elongation in developing styles and produces exserted stigma phenotype; a 450-bp deletion in the *LO2* promoter causes decreased expression of the gene, resulting in short styles of cultivated tomatoes (Chen et al., 2007). In summary, cell sizes and shapes in the style and petal, which are altered by cell division and elongation, are the major determinants of stigma exertion.

Mungbean is an important legume crop in Asia. The crop is well adapted to drought and low-fertility soils, and it matures early (60–75 days). Mungbean seeds are rich in high-quality proteins and starch, which makes mungbean popular with consumers and farmers (Nair et al., 2013; Ebert et al., 2017). However, the major limitation of mungbean production is its low yield. The genetic improvement of mungbean is important to increase mungbean productivity. One strategy that may improve the seed yield of mungbean in commercial production is to use hybrid cultivars (Sorajjapinun and Srinives, 2011). Several studies have suggested that heterosis may be exploited to increase mungbean yield (Barad et al., 2008; Dethe and Patil, 2008; Rout et al., 2010).

Here, we report a novel mungbean mutant, *stigma exposed 1* (*se1*), which affects the development of petals and styles, resulting in wrinkled petals and exserted stigma. The exposed stigma may be useful in promoting outcrossing for the hybrid breeding of mungbean. The objectives of this study were to (i) finely map the *se1* locus and identify candidate gene for this locus, and (ii) molecularly characterize the function of the *se1*.

## Materials and Methods

### Plant Materials and DNA Extraction

Seven mungbean accessions including *se1*, Sulv1, KSP1, KPS2, Jilv7, V2709, and ACC41 were used in this study. *se1*, Sulv1, KSP1, KPS2, Jilv7, and V2709 are cultivated mungbean (*V. radiata* var. *radiata*), whereas ACC41 is wild mungbean (*V. radiata* var. *sublobata*). *se1*, Sulv1, and Jilv7 originated from China; KSP1 and KPS2 originated from Thailand; V2709 originated from India; and ACC41 originated from Australia. All accessions except *se1* are normal mungbean with typical flowers, while *se1* is a mutant mungbean with abnormal flowers including wrinkled petals and exserted stigma. The *se1* mutant, showing wrinkled petals and exserted stigma, used in this study has been initially identified and selected from the M_2_ plant generation of a Sulv1 mutant pool generated by gamma irradiation. A single M_2_ plant with exserted stigma was selected and self-pollinated. Subsequently, a *se1* stable line was continuously selected from M_3_ and M_4_ generations based on floral phenotype. An F_2_ population was developed from the hybridization between V2709 (female) and *se1* (male) for genetic analysis and gene cloning. V2709 is a landrace mungbean with normal flowers. The F_2_ population of 1,535 individuals along with *se1* and V2709 were planted in an experimental field in a cool–dry season (November 2018 to January 2019) at Kasetsart University, Kamphaeng Saen Campus, Nakhon Pathom, Thailand. The distance between plants was 25 cm. The field was clay loam soil with a pH of 6.4. Cultural practices were performed according to Park (1978).

Genomic DNA was extracted from young leaves of F_2_ plants showing exserted stigma and parental plants using a modified CTAB method (Murray and Thompson, 1980).

### Histological Observation

The juvenile flower tissues (0.5–0.8 mm in length) of *se1* and V2709 were collected and fixed in the formalin/acetic acid/alcohol (FAA) fixative [formalin:70% ethanol:acetic acid = 18:1:1 (v/v)]. The samples were placed in a vacuum for 30 min and stored in 70% ethanol at room temperature. The samples were then dehydrated, embedded, trimmed, sectioned, stained, and de-waxed as previously described (Jackson et al., 1994). Sections were observed under an Olympus BX51 microscope (Olympus, Japan).

For observation of the vasculature pattern, 0.5 cm^2^ of the standard tissue was fixed for 4 h in ethanol/acetic acid (6:1) at room temperature. After bleaching in 100% ethanol overnight, the samples were mounted in a mixture of chloralhydrate/glycerol/water (8:1:2) for 1 h at room temperature. Tissues were observed under an Olympus BX51 microscope (Olympus, Japan).

### Segregation Analysis and Fine Mapping of *se1*

To determine the mode of inheritance of exserted stigma, the numbers of mutant and of wild-type plants in the F_2_ population were counted and subjected to a chi-square (χ^2^) test. Initially, the location of the *se1* locus controlling wrinkled petals and exserted stigma (see section “Results”) was identified by genotyping a pooled DNA of 10 F_2_ plants showing the mutant phenotypes with 154 insertion/deletion (InDel) markers (Supplementary Table S1) developed in our previous study (Chen et al., 2016). These markers covered 11 haploid chromosomes of mungbean. Based on the resultant marker genotypes, we focused on a region of chromosome 6 harboring *se1* and then finely mapped this locus by genotyping all the F_2_ plants using new InDel and simple sequence repeat (SSR) markers developed in this study (Supplementary Table S1) which target a 4.632-Mb region (positions 95,497–4,728,487) of mungbean chromosome 11. The SSR markers were developed from the mungbean reference genome sequence (cultivar V1973A)^1^. Primers for SSR were designed using Primer Premier 5.0 software. In case of the InDel markers, primers were developed by comparing sequences of Sulv1 and the reference sequence using mInDel pipeline (Lv et al., 2016). The SSR and InDel markers were screened for polymorphism between the parents.

### PCR and Gel Electrophoresis for Genotypic Analysis

The InDel and SSR markers were detected by polymerase chain reaction (PCR) and gel electrophoresis. For PCR, each 10-μl amplification reaction solution contained 10 mM Tris–HCl (pH 8.3), 50 mM KCl, 1.5 mM MgCl_2_, 50 μM dNTP, 0.2 μM mixed primers, 0.5 U *Taq* polymerase (TaKaRa, Dalian, China), and 20 ng of DNA template. PCR program consisted of a denaturation step (94°C/5 min), followed by 33 cycles of 94°C/30 s, 55°C/30 s, and 72°C/60 s, and a final extension step of 72°C/5 min. The PCR products were electrophoresed through 8% non-denaturing polyacrylamide gels in 0.5 × TBE buffer and bands were visualized by silver staining.

### Genome Re-sequencing

To identify the genome-wide SNPs and InDels between Sulv1 and the *se1* mutant, whole-genome re-sequencing was performed using the Illumina HiSeq 2500 platform in accordance with a previous study (Arai-Kichise et al., 2011). The sequencing depths for Sulv1 and the *se1* mutant were 9.83 and 8.94, respectively. In total, 31,153,168 clean reads, with more than 4.6 billion bases, were generated from Sulv1, and 30,274,654 clean reads, with more than 4.5 billion bases, were generated from *se1*. The mungbean reference genome (Kang et al., 2014) was used as a reference to detect SNPs and InDels between Sulv1 and *se1*, and 1,048,575 SNPs and 86,976 InDels were identified.

### Gene Sequencing

*LOC106777793* encoding a DUF1005 protein was selected as the candidate gene at the *se1* locus (see section “Results”). The gene was amplified using primers listed in Supplementary Table S1. PCR was conducted as described above with an exception that KOD-plus DNA polymerase (Toyobo, China) was used. The DNA bands with expected size were checked on a 1.2% agarose gel, cut, and purified with E.Z.N.A.^®^ Gel Extraction Kit (Omega, Doraville, GA, United States). The purified DNA was sequenced using the BigDye^®^ Terminator v3.1 Cycle Sequencing Kit on an ABI 3730xl DNA Analyzer (Applied Biosystems, Foster City, CA, United States) by Tsingke (Nanjing, China).

### RNA Isolation and Quantitative Real-Time PCR Analysis

Different tissue samples were collected at different stages as follows: roots of young seedlings, leaves and stems (young green leaves and stems from the vegetative growth stage), young flowers (3.0–5.0 mm, with petals shorter than the sepals), mature flowers (7.0–9.0 mm, green flowers before blooming), and inflorescences. Total RNA was extracted using RNA prep Pure plant kit (Tiangen, Beijing, China). One microgram of total RNA was reverse transcribed using cDNA Synthesis Kit with gDNA remover (Tsingke, Beijing, China). Quantitative real-time PCR was performed using an SYBR Premix ExTaqTM kit (Takara, Beijing, China) on an ABI prism 7500 real-time PCR System. *VrACTIN-3* (*Vradi03g00210*) was selected as reference gene. The 2^–ΔΔ*CT*^ method was used to analyze relative gene expression (Livak and Schmittgen, 2001). The gene expression level was calculated from three biological replicates and three technical replicates.

### Phylogenetics Analysis

We determined phylogeny of VrDUF1005. Protein sequences from azuki bean (*Vigna angularis*), cowpea (*Vigna unguiculata*), common bean (*Phaseolus vulgaris*), soybean (*Glycine max*), pigeon pea (*Cajanus cajan*), chickpea (*Cicer arietinum*), Barrel medic (*Medicago truncatula*), Lupin (*Lupinus angustifolius*), Arabidopsis, tobacco (*Nicotiana tabacum*), and maize (*Zea mays*) matching that of VrDUF1005 were identified using the BLASTP algorithm, and aligned with DNAMAN5.2 software (Lynnon Biosoft Corp.). A rooted phylogenetic tree was constructed by neighbor-joining method using MEGA 10.0 software (Kumar et al., 2018). Robustness of the phylogeny was tested by bootstrapping (1,000 replicates).

### Scanning Electron Microscopy (SEM)

Mature petals (standards, wings, and keels) of *se1* and V2709 were fixed in 2.5% v/v glutaraldehyde solution. After dehydration by the ethanol solution series and critical point drying, the materials were coated with gold palladium in E-1010 ion sputter. SEM was performed with a Hitachi S-2460N scanning electron microscope (Hitachi High Tech. Corp., Japan) at 15 kV. SEM photographs were captured electronically and processed with the Adobe Photoshop 7.0 software.

### Subcellular Localization of VrDUF1005

The coding sequence (cds) of *VrDUF1005* was cloned in-frame in the pAN580-GFP [green fluorescent protein (GFP)] vector to create a fusion construct under control of the CaMV 35S promoter. Two fusion vectors, 35S::VrDUF1005-GFP and 35S::GFP-VrDUF1005, were generated and infiltrated into 4-week-old *Nicotiana benthamiana* leaves. P19 was used as a transgenic silencing inhibitor. After 48–72 h, the GFP signal was visualized under the LSM700 confocal laser scanning microscope (Zeiss, Oberkochen, Germany).

### Observation of Pollen Germination in the Stigma

The blooming flowers from the Sulv1, *se1* mutant, and *se1* mutant pollinated with pollens of Sulv1 were collected and fixed in the FAA solution overnight. Other parts of the flower were removed with only pistil left, then washed three times with tap water and softened in 1 M NaOH for 12 h. Subsequently, the samples were gently washed with tap water and stained with 0.1% (w/v) aniline blue in 0.1 M K_2_HPO_4_ for 4 h and incubated in the dark for 20 min. Then, the samples were observed using a fluorescence microscope.

### Arabidopsis Transformation

For the functional analysis, the full-length coding sequence of *VrSE1* was cloned and inserted into the 1,305.1-GFP vector driven by the cauliflower mosaic virus 35S promoter. The plasmid was transformed into the *Agrobacterium tumefaciens* EHA105 strain, and an *Arabidopsis thaliana* (ecotype Col-0) transformation was performed using the floral dipping method as described previously (Qi et al., 2017). The seeds of transgenic plants were selected on 1/2 Murashige and Skoog culture media containing the proper antibiotics.

### Statistical Analysis

Statistically significant differences in the mean values between the wild type and the *se1* mutant were determined by unpaired two-tailed Student’s *t*-test using Microsoft Excel version 2013 software. All the values and data points are presented as means ± SD.

## Results

### Characterization of the *Stigma Exposed 1* Mutant

**FIGURE 1:**
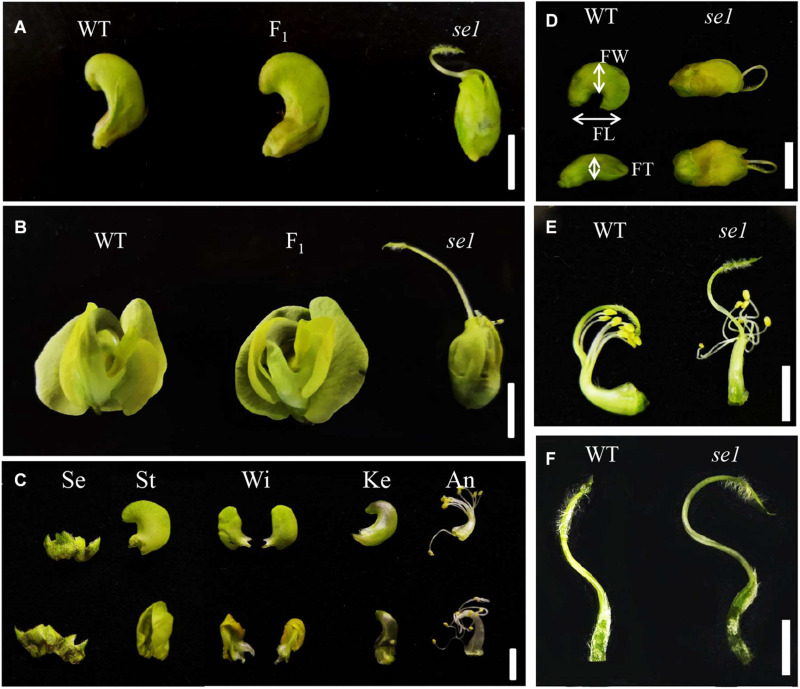
Comparison of floral characters between wild type and *se1* mutant. **(A)** Closed flower of wild type (WT), *se1* mutant, and F_1_ hybrid. **(B)** Blooming flower of wild type (WT), *se1* mutant, and F_1_ hybrid. **(C)** Floral organs in a mature flower of wild type (above) and *se1* (down). Se, sepals; St, standard petals; Wi, wing petals; Ke, keel petals; An, androecium. **(D)** Flower size comparison of wild type (WT) and *se1* mutant. FW, FL, and FT are the abbreviations for flower width, length, and thickness, respectively. **(E)** Fusion stamen and pistil of wild type (WT) and *se1* mutant. **(F)** Pistil of wild type (WT) and se1 mutant. Bars = 1 cm in **(A–D)**; bar = 5 mm in **(E,F)**.

We characterized one mungbean mutant that showed defects in the petal shape and stigma length from Sulv1 mutant library (Figure 1). There were no differences between this mutant and the wild type during the vegetative stage; however, the flowers of the mutant were abnormal. The flowers did not open during the reproductive stage, and most of the stigma were exposed above the petals (Figures 1A,B); consequently, we named this mutant *stigma exposed1* (*se1*). The petals, including standard, wings, and keels of *se1*, showed severe developmental defects (Figure 1C). There was a significant change in the flower size in the *se1* mutant, and the width and the thickness increased (Figure 1D and Table 1). Moreover, although the *se1* mutant produced normal anthers with fertile pollens (Supplementary Figures S1A,B), it produced fewer pods per plant and seeds per pod than the wild type, Sulv1 (Supplementary Figures S1C–H). This was principally the result of the spatial separation between stigma and anthers in *se1* (Figure 1E), stemming from a significant increase in stigma lengths (Figure 1F and Table 1). The characteristics of each floral organ in the *se1* mutant were not changed [sepals, petals, 9 (fused) + 1 (free) stamens and carpel] in four whorls (Figure 1C); therefore, the *se1* mutant displayed specific defects on the late development of petals and stigma.

**TABLE 1:** Comparison of floral characteristics between *se1* mutant and its wild type, Sulv1.

Character	*se1*	Sulv1	*P*-value
Flower length (mm)	9.14 ± 0.12	9.34 ± 0.28	0.17
Flower width (mm)	5.31 ± 0.21	4.43 ± 0.17	2.68E–05
Flower thickness (mm)	5.47 ± 0.29	3.19 ± 0.19	1.06E–07
Stigma length (mm)	14.18 ± 1.16	10.15 ± 0.29	3.93E–05

### Map-Based Cloning of *se1*

The inheritance of the exposed stigma was determined using an F_2_ population generated from a cross between the *se1* mutant and mungbean accession V2709. F_1_ plants showed normal flowers (Figures 1A,B). In the F_2_ population, wild-type and mutant-type plants segregated 1,178:357, corresponding to a 3:1 ratio (χ^2^ = 2.49 < *P*_0.05_ = 3.84). These results indicated that *se1* is a single recessive nuclear gene.

**FIGURE 2:**
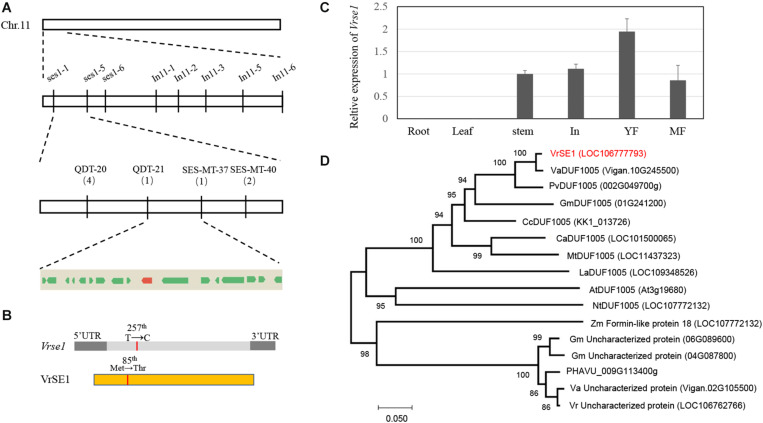
Mapping-based cloning and phylogeny analysis of *se1*. **(A)** Physical map of *se1* on chromosome 11. *Se1* locus is placed in the region of 156 kb, between marker QDT-21 and SES-MT-37; numerals under markers indicate the number of recombinants. Seventeen predicted genes inside this region are indicated by boxes, and the red box indicates *LOC106777793*. **(B)** Schematic structure of VrSE1 gene and protein; dark gray boxes indicate the untranslated region (UTR), light gray box indicates the exon, yellow box indicates the DUF1005 protein, and the red lines indicate the mutation site. **(C)** Transcript levels of *VrSE1* in different tissues of Sulv1. In inflorescences, YF young flowers, MF mature flowers. **(D)** Neighbor-joining tree of DUF1005 family members from *Vigna angularis* (Va), *Vigna unguiculate* (Vu), *Phaseolus vulgaris* (Pv), *Glycine max* (Gm), *Cajanus cajan* (Cc), *Cicer arietinum* (Ca), *Medicago truncatula* (Mt), *Lupinus angustifolius* (La), *Arabidopsis thaliana* (At), *Nicotiana tabacum* (Nt), *Zea mays* (Zm), and *Vigna radiata* (Vr).

Whole-genome scanning using a pooled DNA of 10 individuals having mutant-type flower revealed that InDel markers In11-1 located at position 2,714,704 on chromosome 11 is perfectly associated with the *se1*. This suggested that *se1* is located on chromosome 11 (Figure 2A). Fine mapping conducted using 357 mutant-type plants and additional molecular markers delimited *se1* to a 156 kb region between markers QDT-21 and SES-MT-37 (Figure 2A). There are 17 putative genes in this interval (Figure 2B).

**FIGURE 3:**
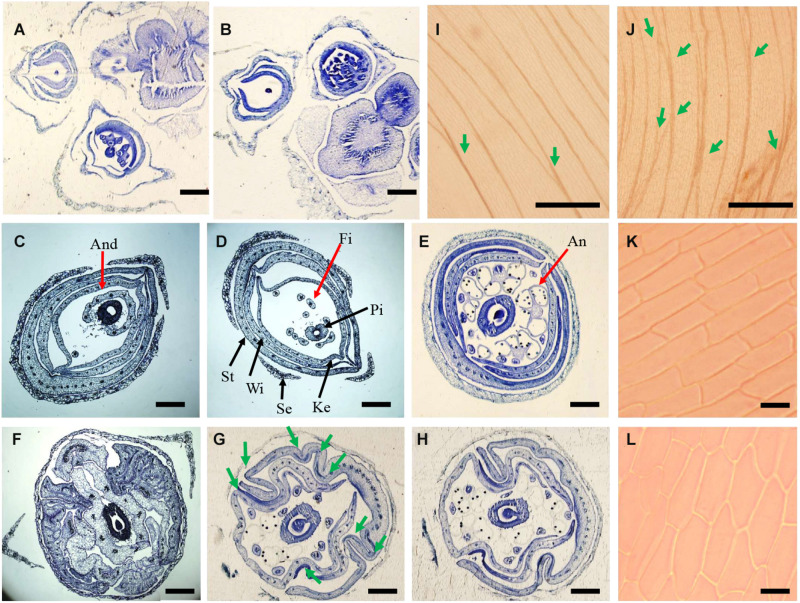
Histological analysis of flowers in wild type and *se1* mutant. **(A,B)** Cross section of inflorescence in wild type **(A)** and *se1* mutant **(B)**. **(C–H)** Cross section and morphology of juvenile flowers in the wild type **(C–E)** and *se1* mutants **(F–H)** at different position: bottom **(C,F)**, middle **(D,G)**, and top **(E,H)**. The red arrows point to the androecium **(C)**, stamen filament **(D)**, and anther **(E)**. The black arrows point to other floral organs, like sepal, standard, wing, keel, and pistil in **(D)**. Green arrows point knotted structures of *se1* mutant flower in **(G)**. And, androecium; Fi, filament; An, anther; St, standard; Wi, wing; Ke, keel; Se, sepal; Pi, pistil. **(I,J)** vasculature in standard of wild type **(I)** and *se1* mutant **(J)**. Green arrows indicate the cross of veins. **(K,L)** Cellular morphology of adaxial epidermal cell of wild type **(K)** and *se1* mutant **(L)**. Bars = 120 μm in **(A,B)**; bars = 100 μm in **(C–H)**; bars = 150 μm in **(I,J)**; bars = 10 μm in **(K,L)**.

Re-sequencing of the genomic DNA of Sulv1 and *se1* led to the detection of 190 SNPs (Supplementary Table S2) and 25 InDels (Supplementary Table S3) in the mapping region. Only five SNPs led to non-synonymous mutations, which were distributed in five genes (Supplementary Table S2). Among these five SNPs, three in *se1* had the same bases as the reference genome; consequently, we focused on the remaining two located in genes *LOC106777216* and *LOC106777793*. According to the PFAM database^2^, *LOC106777216* encodes a SOSS complex subunit B homolog (data not shown), while *LOC106777793*, having only one exon, encodes a DUF1005 domain-containing protein (Figure 3B). Because *LOC106777793* showed high homology levels with genes encoding formin-like proteins (Figure 2D), which are involved in cell division and expansion, we considered *LOC106777793* as the candidate gene of the *se1*. qRT-PCR showed that *LOC106777793* highly expressed in young flower of mungbean (Figure 2C). Furthermore, sequencing revealed a single base substitution in the encoding region, causing amino acid change in *LOC106777793* (Figure 2B). The amino acid change was methionine in the wild type to threonine in the mutant. qRT-PCR results indicated that *Vrse1* only expressed in stem and reproduction organs, and the expression level increased during the development of flower (Figure 2C). Further, we sequenced this gene in different mungbean lines (KPS1, KPS2, jilv7, and ACC41) possessing normal flowers. Sequence alignment confirmed that the 1 bp substitution occurred only in the *se1* mutant (Supplementary Figure S2A). Taken together, these results demonstrated that *LOC106777793* was responsible for the phenotype of the *se1* mutant.

### Phylogenetic Analysis of SE1

We analyzed orthologous proteins of DUF1005 in different species (Figure 2D). This protein is highly conserved in higher plants and found in both dicots and monocots. There are five homologous proteins in mungbean (Supplementary Figure S2B), but they are lowly conserved (less than 50% identity). The most homologous protein came from Phaseoleae species including azuki bean (98.5% identity), cowpea (91.6% identity), and soybean (81.1% identity). The protein is highly conserved in legume plants, including chickpea, pigeon pea, Medicago, and lupin, and these phylogenetic relationships corresponded to the evolutionary route in the Leguminosae (Azani et al., 2017). Interestingly, the DUF1005 family of protein was not found in members of the tribe Viceae, including fava bean, lentil, and pea. This suggested that there may have been a whole-gene deletion during the diversification of the tribe Viceae.

Because there is no significant signal peptide predicted in SE1 protein, two fusion proteins, VrDUF1005-GFP and GFP-VrDUF1005, were constructed to examine the subcellular localization of the VrDUF1005. The GFP fluorescent signals were detected in both nuclei and plasma membranes (Supplementary Figure S2C), suggesting that DUF1005 localizes to both the membrane and nucleus, but based on fluorescence intensity, it is mainly located on cell membrane.

### SE1 Affects Cell Shape in Petals and Cell Length in Stigma

**FIGURE 4:**
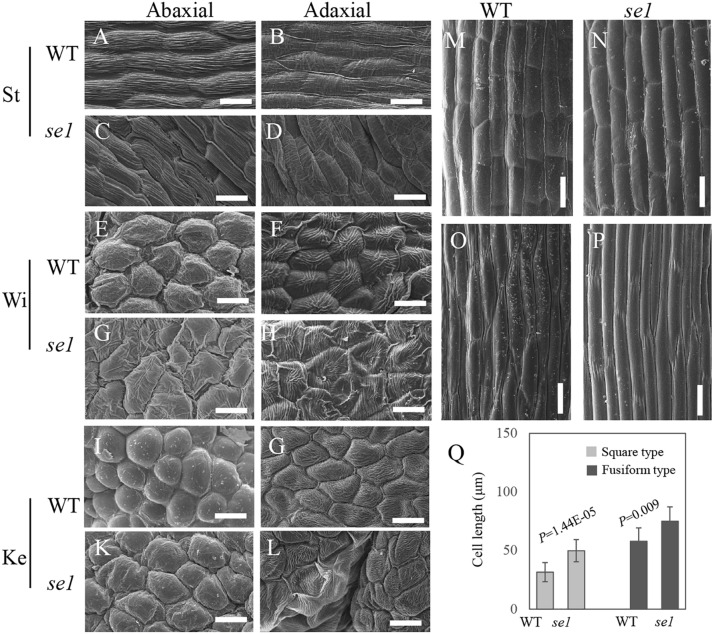
The scanning electron microscopy (SEM) analysis of epidermal cell of petals and stigma in wild type and *se1* mutant. **(A–D)** Cellular morphology of abaxial **(A,C)** and adaxial **(B,D)** epidermal layer in standard of wild type **(A,B)** and *se1* mutant **(C,D)**. **(E–H)** Cellular morphology of abaxial **(E,G)** and adaxial **(F,H)** epidermal layer in standard of wild type **(E,F)** and *se1* mutant **(G,H)**. **(I–L)** Cellular morphology of abaxial **(I,K)** and adaxial **(G,L)** epidermal layer in standard of wild type **(I,G)** and *se1* mutant **(K,L)**. **(M–P)** Cellular morphology of square **(M,N)** and fusiform **(O,P)** cell in styles of wild type **(M,O)** and *se1* mutant **(N,P)**. **(Q)** Cell lengths in style of wild type and *se1* mutant. Values are means ± SE, statistical significance was determined by the Student’s *t*-test, Bars = 20 μm in **(A–L)**, Bars = 15 μm in **(M–P)**.

A histological study was conducted to investigate the wrinkled petals and longer stigma in the *se1* mutant. Transversal cross sections of young inflorescence showed no significant differences between the wild type and the *se1* mutant (Figures 3A,B). Cross sections of young flower (5–7 mm in length) were taken from three different positions along the flower (bottom, middle, and top). Based on the observation of androecium, filaments, and anthers, the petal arrangement was not altered in the *se1* mutant. Standard, wing, and keel petals were arranged in order from outside to inside and still maintained their symmetry along the dorsoventral axis. The petals displayed conspicuous irregular bending and curvatures with noticeable knotted structures, and the standard did not cover the keels (Figures 3C–H). In addition, the longitudinal main vein pattern of the wild-type standard was more parallel with limited cross numbers; however, there were more crosses between contiguous veins (Figures 3I,J). Moreover, the shape of epidermal cells in standard petal of the *se1* mutant had changed (Figures 3K,L). To further explore the differences in floral cells between the wild type and the *se1* mutant, a close examination of outer and inner epidermal cells was performed by using SEM. The surfaces of all the petals were smooth in the wild type but rough in the *se1* mutant. Representative cells were recognized in each petal by specific characteristics, but the epidermal cell shape in both the adaxial and abaxial layers was irregular in the *se1* mutant (Figures 4A–L). We noticed that there are two types of cells in mungbean styles, square (near the ovary), and fusiform (near the stigma); neither type displayed a change in shape in the *se1* mutant flower, but the cell lengths of both types were significantly increased (Figure 4Q). These data suggest that the development of petals and stigma in the *se1* mutant is affected at the cellular level and that the processes of petal cell division and stigma cell elongation may have malfunctioned.

### Overexpression of *VrDUF1005* in Arabidopsis

**FIGURE 5:**
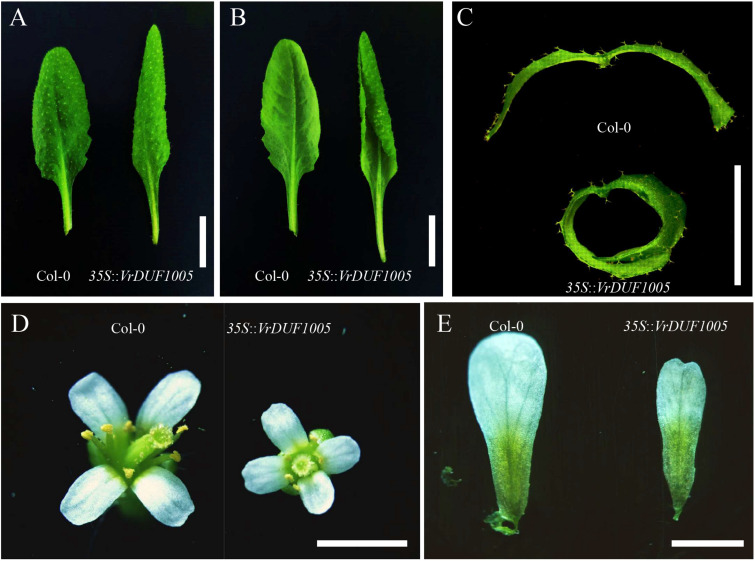
Phenotype of transgenic *Arabidopsis of 35S::VrDUF1005.* **(A,B)** Leaf morphology of Col-0 and overexpression line of *35S::VrDUF1005*. **(C)** Leaf hand cross section of Col-0 and overexpression line of *35S::VrDUF1005*. **(D)** Floral morphology of Col-0 and overexpression line of *35S::VrDUF1005*. **(E)** Petals of Col-0 and overexpression line of *35S::VrDUF1005*. Bars = 1 cm in **(A,B)**; bars = 5 mm in **(C,D)**; bars = 2 mm in **(E)**.

To study the function of VrDUF1005, we ectopically expressed *VrDUF1005* gene in Arabidopsis driven by the cauliflower mosaic virus 35S promoter. Three independent overexpression lines were obtained, and we investigated the phenotypes in the T_2_ generation; the rosette leaves of the positive transgenic plants were rolling (Figures 5A–C), and the flower size reduced (Figures 5D,E). Since these phenotypes were related to cell shape and size, the results from transgenic analysis corroborated that VrDUF1005 regulates cell shape and cell size.

### Fertility Investigation of the *se1* Mutant

**FIGURE 6:**
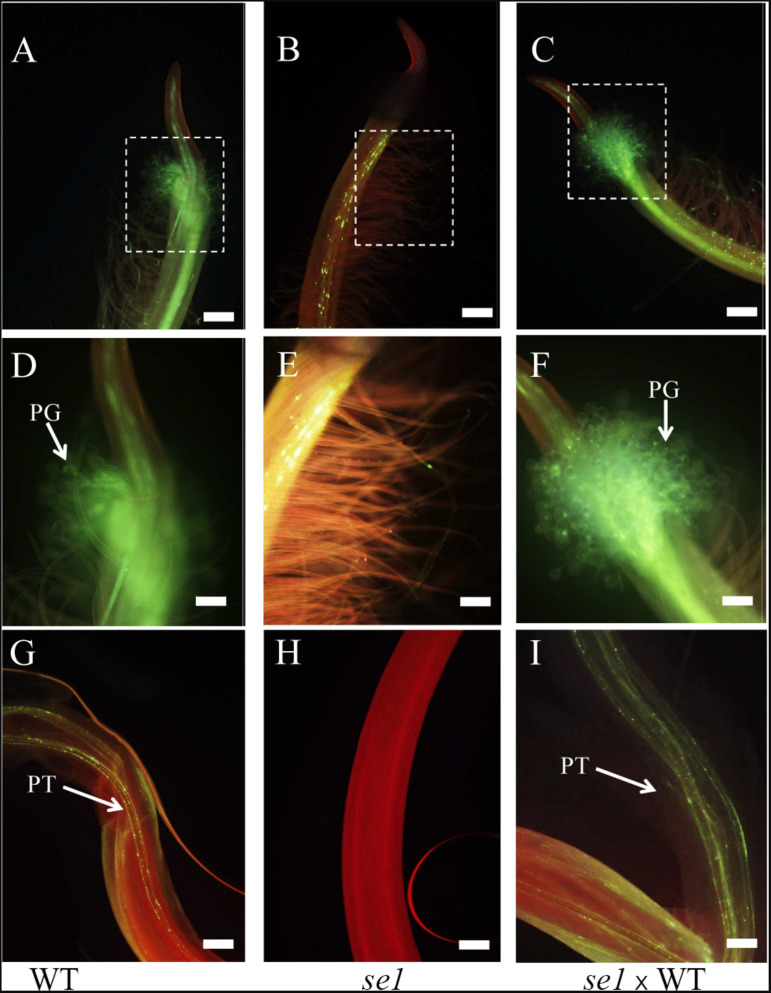
Aniline blue staining of germinated pollen and pollen tubes growing in planta. **(A–C)** Pistils of natural self-pollinated wild type **(A)**, natural grown *se1* mutant **(B)**, and *se1* mutant pollinated by wild-type pollens artificially **(C)**. **(D–F)** The enlarged images of stigmas correspond to the dotted boxed areas in **(A–C)**. PG, pollen grain. White arrows indicate the trajectory of pollen grains. **(G–I)** Pollen tubes grow in style of natural self-pollinated wild type **(G)**, natural grown *se1* mutant **(H)**, and *se1* mutant pollinated by wild-type pollens artificially **(I)**. PT, pollen tube. White arrows indicate the trajectory of pollen tubes. Bars = 200 μm in **(A–C,G–I)**; bars = 100 μm in **(D–F)**.

Approach herkogamy is an ideal floral architecture to develop hybrid breeding of autogamous crops (Webb and Lloyd, 1986). To assess the potential of the *se1* mutant for mungbean hybrid breeding, we investigated the fertility of pollen grains on the stigma. The pollen grains germinated on the stigma of natural selfing wild-type plants (Figures 6A,D); the pollen tubes were able to grow and elongate along the style (Figure 6G). However, there were no pollen grains on the exposed stigma of the *se1* mutant under natural conditions (Figures 6B,E,H). When pollens of the wild type were artificially transferred to the stigma of the *se1* mutant, these pollens germinated, and the pollen tubes grew normally (Figures 6C,F,I) as the elongated style did not prevent the pollen tube from advancing. Because the *se1* mutant was able to produce pods (Supplementary Figure S1), which is the prerequisite for hybrid breeding, and its exposed stigma prevented it from self-crossing, it appears that the *se1* mutant may be utilized as a female parent in mungbean hybrid breeding without manual emasculation.

## Discussion

Flowers with exposed stigma increase the outcrossing rate and are useful in developing improved hybrid crop cultivars, especially self-pollinated crops. Mungbean is a chasmogamous crop. In this study, we found that exposed stigma in *se1* mutant mungbean is controlled by a single recessive gene. We successfully finely mapped the gene, *se1*, and identified *VrDUF1005* as the candidate gene for the trait. We further characterized the function of the VrDUF1005.

### VrSE1 Is a DUF1005 Domain-Containing Protein and May Regulate Cell Shape in Mungbean Flower

The ABC model fully explains the specification of floral organs and is widely applied in flowering plants (Coen and Meyerowitz, 1991). In contrast, the late development of floral organs remains poorly elucidated. Petal or stigma organogenesis is determined by cell proliferation and cell expansion after the initiation of the primordium, a process in which auxin plays a key role (Sauret-Gueto et al., 2013; Dang et al., 2020). The genes downstream of auxin signaling promote cell division and expansion by triggering cell-cycle activity (Randall et al., 2015) and regulate cytoskeleton formation (Yang et al., 2019). In this study, we identified a novel gene *VrSE1* that alters petal and stigma development. Petals of the *se1* mutant were wrinkled and the stigma was exposed, while floral organ number was not changed (Figures 1A–C) and the representative epidermal cells were found in different petals of the *se1* mutant (Figures 4A–V). *VrSE1* expressed highly in reproduction organs especially in young flowers (Figure 2C), and these results suggested that *Vrse1* functions in the late development stage rather than the earlier initiation stage. The deformed cell shape in petals and the increased cell length of stigma in the *se1* mutant (Figure 4) indicated that *VrSE1* is a pleiotropic gene, which affects cell division in petals and cell elongation in stigma.

VrSE1 is a DUF1005 family protein that is highly conserved in plants (Figure 3C). Some DUF domain-containing protein appears to be involved in the late development of floral organs. DUF640 is a transcription factor that regulates the late development of lemma and palea in rice (Yoshida et al., 2009; Ma et al., 2013). The REL2 protein, containing DUF630 and DUF632 domains, controls bulliform cell division in rice (Yang et al., 2016). The DUF593 domain-containing protein LLP13 regulates the dynamics and organization of cytoskeleton in pollen tubes of lily (Wang et al., 2014). Nonetheless, DUF1005 shares a very low identity with proteins mentioned above. Interestingly, VrDUF1005 showed high homology level with LOC100273612 in maize and At4g29310 in Arabidopsis. LOC100273612 and At4g29310 were annotated as formin-like protein 18 (Figure 2D). Formin is an essential regulator of cytoskeleton and directly controls cell division and expansion (Cao et al., 2016; Cvrckova et al., 2016; Rosero et al., 2016). However, DUF1005 did not possess the basic FH2 (Formin Homology 2) domain of formin-like protein (Dominguez, 2009). The overexpression of the ectopically expressed *VrSE1*, driven by the 35S promoter in Arabidopsis, displayed rolling leaves and smaller flowers, and such phenotypes may be caused by the change of cell shape and cell size; however, the molecular function of *VrSE1* needs to be further explored. In the *se1* mutant, malfunction in flower development happened after the formation of all petals (Figures 3C,D), and *VrSE1* showed the highest expression level in young flowers (Figure 2C), indicating that the *VrSE1* functions at late development stage after the determination of floral organ identities. Because of its specific effects on petals (whorl 2) and pistils (whorl 4) (Figure 4), *VrSE1* is most likely a target or downstream gene of E-class gene (Coen and Meyerowitz, 1991) that is involved in mungbean flower development. However, the molecular function of *VrSE1* needs to be further explored.

### The *se1* Mutant as a Resource for Mungbean Hybrid Breeding

In the field, the *se1* mutant displayed an exposed stigma, while the anthers were still enclosed inside the keels (Figure 1); this spatial separation prevents self-crossing, resulting in a very low pod setting rate and short pod in the *se1* mutant (Supplementary Figure S1). However, the elongated pistil did not affect the biological function of stigma as artificial pollination showed that the foreign pollens can germinate on the stigma and the pollen tubes extended through the style (Figure 6). Thus, the *se1* mutant may be used as a female parent to accept pollen grains from other varieties without emasculation. Male-sterile female line(s) with CMS and nuclear-controlled environment-sensitive genic male sterility (EGMS) are pivotal resources in hybrid cultivar production (Chen and Liu, 2014). Nevertheless, the application of CMS lines in hybrid breeding of legume crops had limited success. For mungbean, no CMS line has been reported to date (Bohra et al., 2016). The only example of large-scale hybrid seed commercialization was in pigeon pea (Saxena et al., 2005) through the development of a CMS line by interspecies hybridization. Meanwhile, our study provides an alternative for mungbean hybrid breeding.

## Data Availability Statement

The raw data supporting the conclusions of this article will be made available by the authors, without undue reservation. Genome sequence data of Sulv1 and se1 mutant are deposit at GenBank (https://www.ncbi.nlm.nih.gov) (accession PRJNA674946).

## Author Contributions

XC and PS conceptualized, supervised the research, reviewed, and revised the manuscript. YL, PS, and XC designed the study. XC secured research funds. PS, KL, and KA developed the population. YL, KL, JC, XY, and KA performed gene mapping and sequencing. YL, RW, and KL performed cytology experiment and transformation. YL and PS analyzed the data and prepared the manuscript. All authors contributed to the article and approved the submitted version.

## Conflict of Interest

The authors declare that the research was conducted in the absence of any commercial or financial relationships that could be construed as a potential conflict of interest.

## Footnotes

1 http://plantgenomics.snu.ac.kr

2 www.sanger.ac.uk/cgi-bin/Pfam

## Supplementary Material

Supplementary Figure 1 Fertility investigation of wild type and *se1* mutant **(A,B)** Pollen iodine staining of wild type **(A)** and *se1* mutant **(B)**. **(C,D)** Pod setting in the field of wild type **(C)** and *se1* mutant **(D)**. **(E,F)** Pod **(E)** and seed setting in the pod **(F)** of wild type (Sulv1) and *se1* mutant. **(G,H)** Pod number per plant **(G)** and seed number per pod **(H)** of wild type (WT) and *se1* mutant. Values are means ± SE, statistical significance was determined by the Student’s *t*-test. Bars = 20 μm in **(E,F)**. Bars = 3 cm in **(E,F)**.

Supplementary Figure 2 Sequence analysis of *VrSE1* in mungbean. **(A)** Sequence alignment of *VrSE1* coding region in different mungbean lines (*se1* mutant, KPS1, KPS2, jilv7 and ACC41), the substitution is indicated in pink color. **(B)** Neighbor-joining tree of DUF1005 family members in mungbean. **(C)** Intracellular localization of VrSE1 in *Nicotiana benthamiana* leaves. GFP, fluorescence of the fusion protein; Nucleus, fluorescence of nucleus marker; Bright, bright-field; Merged, overlay of previous images. Bars = 30 μm.
