# Supplementary material for: Mapping and Functional Characterization of Stigma Exposed 1, a DUF1005 Gene Controlling Petal and Stigma Cells in Mungbean (Vigna radiata)
Source: Front Plant Sci. 2020 Nov 19;11:575922. doi: 10.3389/fpls.2020.575922 (PMC7710877; doi:10.3389/fpls.2020.575922)

Supplementary Figure S1. Fertility investigation of wild type and *se1* mutant
(A, B) Pollen iodine staining of wild type (A) and *se1* mutant (B). **(C, D)** Pod setting in the field of wild type (C) and *se1* mutant (D). **(E, F)** Pod (E) and seed setting in the pod (F) of wild type (Sulv1) and *se1* mutant. **(G, H)** Pod number per plant (G) and seed number per pod (H) of wild type (WT) and *se1* mutant. Values are means ± SE, statistical significance was determined by the Student's *t*-test. Bars=20 μm in (E, F). Bars=3 cm in (E, F).

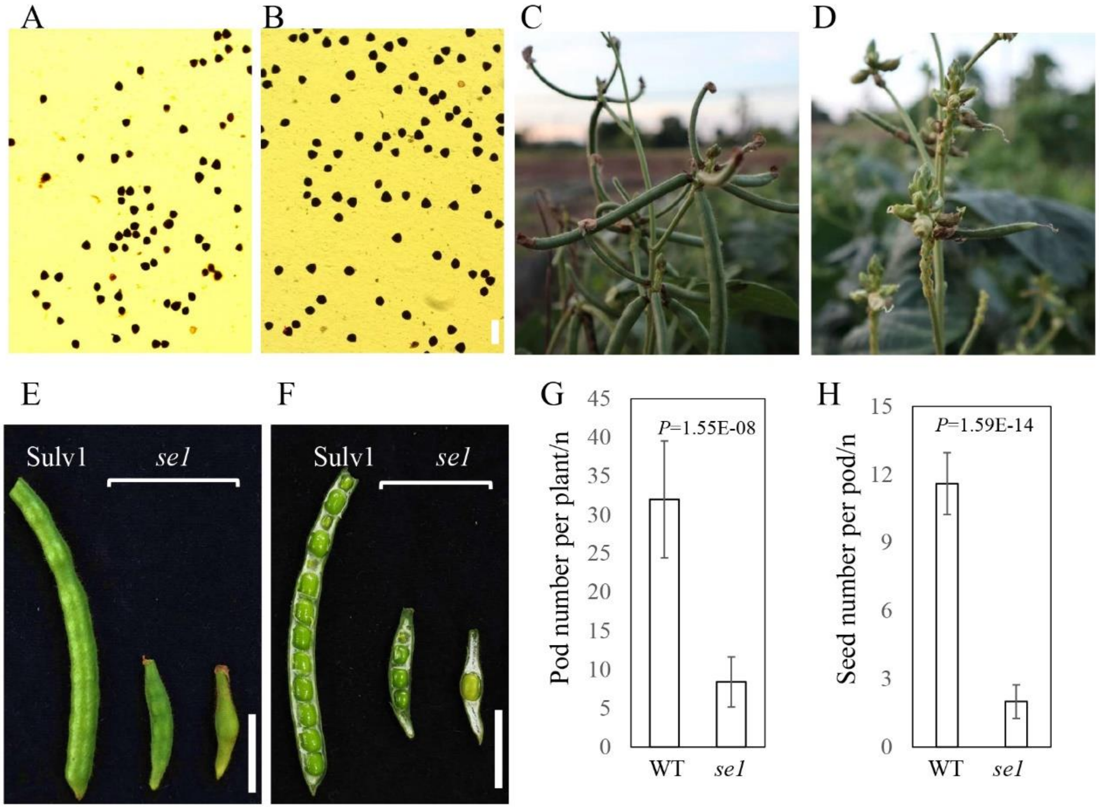

Supplement: Supplementary Figure 1 — Fertility investigation of wild type and se1 mutant (A,B) Pollen iodine staining of wild type (A) and se1 mutant (B). (C,D) Pod setting in the field of wild type (C) and se1 mutant (D). (E,F) Pod (E) and seed setting in the pod (F) of wild type (Sulv1) and se1 mutant. (G,H) Pod number per plant (G) and seed number per pod (H) of wild type (WT) and se1 mutant. Values are means ± SE, statistical significance was determined by the Student’s t-test. Bars = 20 μm in (E,F). Bars = 3 cm in (E,F). [file Data_Sheet_4.PDF]
