# Supplementary material for: Mapping and Functional Characterization of Stigma Exposed 1, a DUF1005 Gene Controlling Petal and Stigma Cells in Mungbean (Vigna radiata)
Source: Front Plant Sci. 2020 Nov 19;11:575922. doi: 10.3389/fpls.2020.575922 (PMC7710877; doi:10.3389/fpls.2020.575922)

Supplementary Figure S2. Sequence analysis of *VrSE1* in mungbean. **(A)** Sequence alignment of *VrSE1* coding region in different mungbean lines (*se1* mutant, KPS1, KPS2, jilv7 and ACC41), the substitution is indicated in pink color. **(B)** Neighbor-joining tree of DUF1005 family members in mungbean. **(C)** Intracellular localization of VrDUF1005 in *Nicotiana benthamiana* leaves. GFP, fluorescence of the fusion protein; Nucleus, fluorescence of nucleus marker; Bright, bright-field; Merged, overlay of previous images. Bars=30 μm.

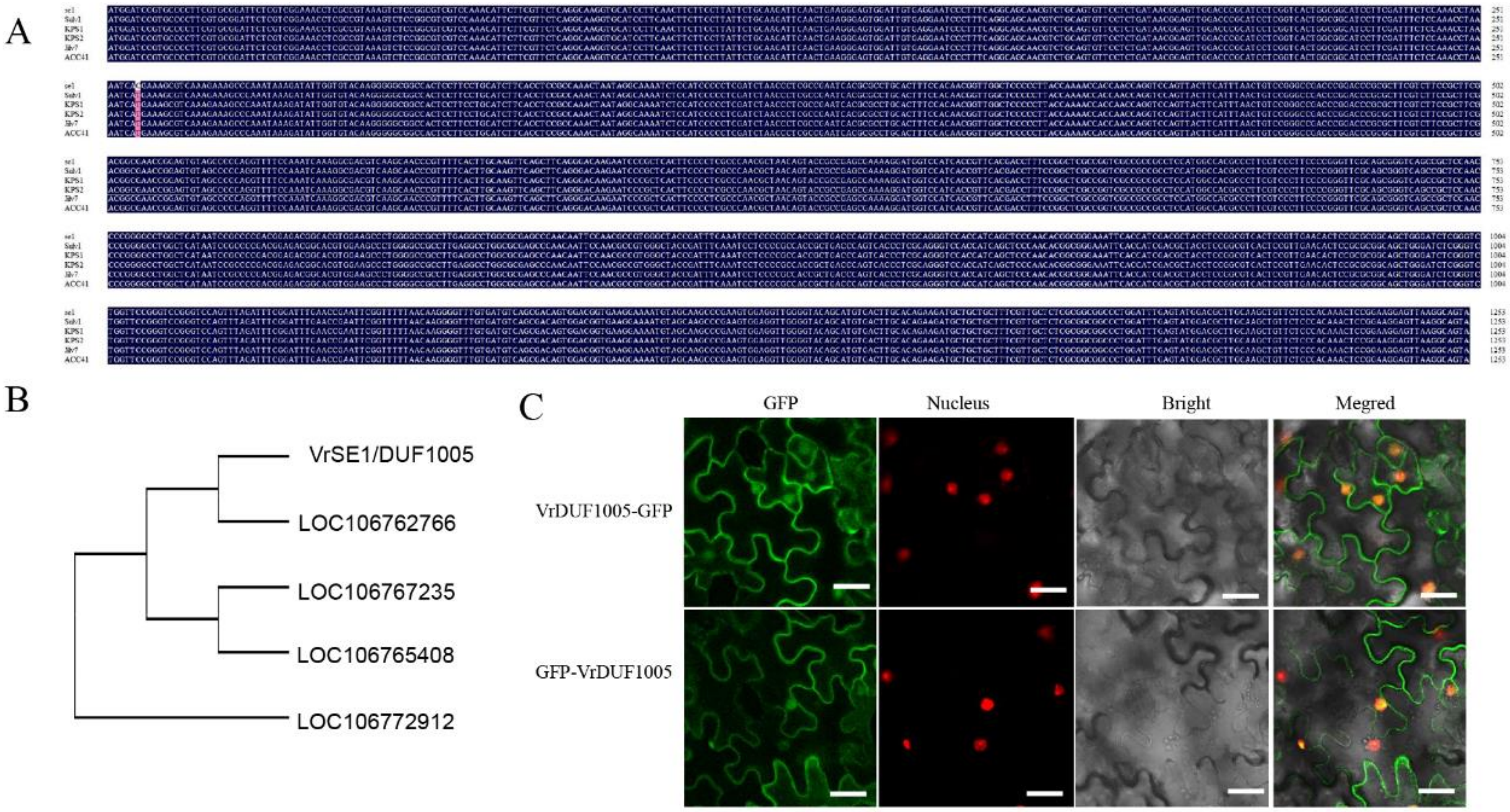

Supplement: Supplementary Figure 2 — Sequence analysis of VrSE1 in mungbean. (A) Sequence alignment of VrSE1 coding region in different mungbean lines (se1 mutant, KPS1, KPS2, jilv7 and ACC41), the substitution is indicated in pink color. (B) Neighbor-joining tree of DUF1005 family members in mungbean. (C) Intracellular localization of VrSE1 in Nicotiana benthamiana leaves. GFP, fluorescence of the fusion protein; Nucleus, fluorescence of nucleus marker; Bright, bright-field; Merged, overlay of previous images. Bars = 30 μm. [file Data_Sheet_5.PDF]
