# Supplementary material for: Mapping and Functional Characterization of Stigma Exposed 1, a DUF1005 Gene Controlling Petal and Stigma Cells in Mungbean (Vigna radiata)
Source: Front Plant Sci. 2020 Nov 19;11:575922. doi: 10.3389/fpls.2020.575922 (PMC7710877; doi:10.3389/fpls.2020.575922)
Supplement: Supplementary file 3 [file Data_Sheet_1.PDF]

Supplementary Table S1 Primers used in this study.

InDel markers		
**Marker name**	**Forward sequence**	**Reverse sequence**
In1-1	TTCATTGGATTCCTTTCAGCA	CATCTAACAAATGCATCGCG
In1-2	CCAATTGTCAAAGTCAGGTCA	GCGGAGATCGACAAGAAAAC
In1-3	TGAACTAAGACAAAGCGCGA	CGGTTCCACTTTTGATCGTT
In1-4	AATCAGGTACAGCAAAAGAAAAA	GTAACGTGGTCCAGGGAAAA
In1-5	CCTTGTTTGTTTTCGGACAAA	GAGAACGGAAGAGACACGGA
In1-6	GATGACGCCTTGGTGTTTTT	GCAGCCTCAAGAGCTTTGAT
In1-7	TGGATTGAGGCAAATTTTAAAAA	CTCTCGAACCCATTGGAAAA
In1-8	CCCAAATTGCCTAATTTATCC	CGATGAAATATTGATCCACGA
In1-9	CATGTGAGATCAATGGTGCC	TTCCCAGCTTGGTTCAACTT
In1-10	GGGGGAAGAAAAGACAGACA	TGGCCCAATATACAAGGCTC
In1-11	CGAAGATGAAACCCTTACCG	TCCAGGCATAAGGTTTTTGC
In1-12	TGTGGTCATTCTCTGCAACTTT	TGTTGTTGTGGGAGTTGTCTG
In1-13	TGATCCCGTGCAACCTTTA	CCTCCAGGTCATTGCTGAAT
In1-14	TCACATGTCATGTTTTCATTGG	TCACGTCTTAAAGGTCAAACTGA
In1-15	TTGAAGCTGGAACATGTTAAGC	AGAAAAGTGTGTGCAAAAGATTGT
In2-1	ACCCAGAGGTGGTGGTTATG	TAAGCTGGCTTCTTCCCTTG
In2-2	TGCTTCAAGGGTGACATCAA	GGTTCGCCATTGTCATCTTT
In2-3	ATTTCCTGTTGTTTGCTGGG	TTTTCTCACTTGCATACGCTGT
In2-4	GAAAGGCACCAAAACCAAAC	TGCCTTAAACCAGAGAATGACA
In2-5	CCTTCAAGTCACCATGTCCC	TTGATTCCTTGCAGAGCAAA
In2-6	AGTGAAGTTCTGCCCTGGTG	CAAAATAGCCGGCAATGTTT
In2-7	CGTATCCCTGCATCTCCTTC	TTCCAGTTTGCCCAACCTAC
In2-8	CGTTCTCATCCACAGCTTCA	GGAATGAAACGAACATGGCT
In2-9	CAGAAAAGCTGGGCAAGTTC	ATCTCCACATACCTCGCAGG
In2-10	TCACCCAAAAGAATGCAACA	TTGGCCTGAGAATGCTTGAT
In2-11	TGCACTGATGATGATGCAGA	ATGGTGTACCTTGCATGCAA
In2-12	GGAAGTGAGTTCCTTGTTGGA	GATTAGTCTGTTTCACGTCTTGGAT
In3-1	CATAAGGTGAGCCCAATTCAA	TTCAATGGTTCTACGCCATG
In3-2	GAAGCAGGAAGGAAAGAGGG	TGTCTATTTTCCAGGCGTATCA

In3-3	ACAAGCACAACGACAGCAAC	ACAATTCGAATTTCCGCAAA
In3-4	CCCCGTTCCAAAATAGGATAA	TGCGCAGATATTCGTGGATA
In3-5	TGATACCGAGACCCGACTCT	TTAAAATGGGGTTCGGCC
In3-6	TGTGAATGGTGGGTCAACAT	TCCTGAGCCCAAAAATCTTG
In3-7	AGCAAGTGACACATGACACTCA	CACCCTGGGTGTCGAGAT
In3-8	AGGATGTTTTTGTGTCCCCT	GGGACAGAGCTATGCAAAGG
In3-9	TTCCGACGATCACTGAGTCA	TGTAAAGTGTCGGACGTTGAA
In3-10	AAGCTGACAGTGACACACGC	TCGATGGTTGAAATGCAATT
In3-11	GTCCATATCAGCAATGGCAA	GGGGTCTTTTTGTTAATGCC
In3-12	AAATCATACCCCTTCCGACC	TCCCGAACGTGGACTTCTAT
In3-13	CACCCAAAATACAACAGAATCATC	ACCATCCCATCTTTTCCTCC
In3-14	GCGGCTCTTTTCTTAAAGCA	CAGCCATGTCAATATGCAGC
In4-1	GCTTTAGGGGGAAGCACTTC	GCCCTCGAGAATCATTTTACA
In4-2	CGGTTGCGACAAAAGTGTAA	TTCATTGGCCAGATTCATCA
In4-3	AGTGACTGGGTGACGGTGAT	ACCCGATCTATTTAGAAGCGC
In4-4	CCTTCTGTCCCACTGCATTT	ATGCGTTAATCAGCAGACTTTG
In4-5	TGGCCAATTTTCGTGTATTG	GGCATGTAATTGATGAAACCAA
In4-6	ACTTCACCGTCACAACTCCC	CAAGCAAACCCAAAAGCTTC
In4-7	TGACAAATCATACCCTGTGCA	TTGTTTCTAGCCAAAACACAAA
In4-8	AATGGAGAACCCCTAAAGGC	TGCCAGAAATGAACAGTCCA
In4-9	GCATGAATATTGTAGCATTCATCA	TTCTGCAACCCTTTTGTTTTCT
In5-1	TGGTGTTTAGTCTGAGTGTCCG	GTGTGTCCAAGCTTCATAGCA
In5-2	TCTATTGGGCGAAAATCCTTT	ACCACTTGCAAAGCCTGAGT
In5-3	GCCAAACCGAACAATGATTT	GCAGTGTCATTTCGGAAAAG
In5-4	AAAGTTCGGTTTCAGCTCGA	AGGAATGCTCACAACTTTCAA
In5-5	GAGGCTACTCGTGCGCTAAA	CATTGCTGGGATTGGACTCT
In5-6	TGTTGAACCACATTAAATCAATGTC	CTCTAGCCACCAAAGCTCATC
In5-7	TTTTTGCATGTCCTACGTCTTG	AAAAAGGCCGGAGAGGAAG
In5-8	AAAATTGCAGCCTTTTCCCT	TGAACCATTTGGGCATATCA
In5-9	AAGCTATGAGACCTACGCGC	GTGATTGGGCTTAAACCGAG
In5-10	CATGTTGCACCTGGAAATTG	TCCCTCAAGTCGGTGCATAT
In5-11	CATTCTAGGGGTGCCAACAT	ACTCCCACTTAGGCAAGGAAA
In5-12	ATCTGTCACCCCTTCTTCCC	CGTGCATGATCCACTTGTTC

In5-13	AGCATCCAAACCAAACAACA	ACTCAGCCGCTTCAATGTTT
In5-14	CCGATTGCTCTCAGCTTTTC	CCCACTGTCTCTGCAACTGA
In5-15	TCCAATCTCCCTTACCCTCA	TCAAATTGCATGACAACCAAA
In5-16	TGCAATGTAAAGCTCCACAAA	CAATTTGAGGTGATGTTTGTCTG
In5-17	TTCCACGCTCTATGTCTCCC	CATCACGCCCAAGCTAGAAT
In5-18	ACTCCGTGCAACCATCTTCT	GTTACCCAACGCTGTGATCA
In5-19	GAACCCTCCCCCAACCTATA	TCAATCCCGTACAAATGGCT
In6-1	TTCAATTTTGCATCTAAATCTACG	CCATCGTTTCTGCGGATAAT
In6-2	TCCAATAACAACATTACAATGAATGA	TCATTTCATGTCACTTGAAGATCA
In6-3	AGCTGACCAAATTGGAGACG	ATTATAAGGGGAGCGAGATGC
In6-4	CAACCTACAAAACCACCCCA	TGCATGGTTGGTTGTGGTAT
In6-5	TGGAAAATGAAGGCTCCAAT	ACATGGTATTTCGCCGAGAA
In6-6	TGGAAACCTTTTTGCAAACA	GTAGCTCGGTGGAGTGAACC
In6-7	GTCAGCATTGTCATTGACTTCC	ACCCTCTCACACCCATGAAT
In6-8	TGAGGAATCGCCCTTTAATG	CCAACTCAAATGGAGAGCAA
In6-9	TCGGTGATGTACTTACCCCC	ACATTCATTGCAGTTGCCAA
In6-10	AACCAATTTGTCCATTGTTTCC	CAACAATTATGAACACAGTCCATG
In6-11	TGGTGATGACCCTTGTGTTG	TTTCCTGTAATCTTGAGTTCCCA
In7-1	CCTAATTTTTCTTCCTGCCG	CTTGCATCCCCACAAAAGTT
In7-2	CGTTCGGTTTTCCCATTTTA	TCCATGATTTGGTATGCAGG
In7-3	TGTGTTGGTTGTGTTGAGGTT	TGACAAATTTGGATGGTCCA
In7-4	TGGCATTCTAAGCCATTTCA	AATGGGTGGAATTTTATGGG
In7-5	GATTTACCCACGGCCATAATC	CGACCTTTGGCTTGCATTAC
In7-6	GCCCTGTCTTTCCTAAAGCC	GTCCATGGCTAACCCAAACA
In7-7	GGGGAAGTGTCAAGCTTTTC	AAAATTTTGTTGCCGATTGC
In7-8	TGGATTCATTTAATGGCACTGA	CGGTTCGTTTCTTGAGGGTA
In7-9	GCCCAACAACTGAAAGCAAT	TTTCCGTCTTTTCTCTCTCGA
In7-10	TTCGTTGTTATGAAGTGTGTGG	AGTCAAACATCTTAACTTCCCCTT
In7-11	GCCTCAACCGACTCCTATGA	AATTGGAATCAGAGAAGGCAAA
In7-12	TTCCTCTACACAACAATAACGGT	CTTGGTAACGGTCCTCCTCC
In7-13	TTGTCTCAAATTGACTCGATCA	AACCGTTCGGTTTGTTCGTA
In7-14	GACATGATCGAAACATCGACC	TGATGTTTGTGGGTTTCAGG
In7-15	GCCTCCCCACAAATTCATTA	TGAATTCCATGCATGGCTT

In7-16	TGCGAATATTCTGCTATGCAA	ATCCCTTGGAATGTATTCGC
In7-17	GTTCCCGCAGCAAATAAAGA	CGCAGCACTTGACATCAGAT
In7-18	TAGTCCGCCCCTTTACAAGA	GCCGTTGAATGGATTGAATT
In7-19	AACTTACAAGCCCACGTTGC	GTGTAGAAAGGAGCCATGGG
In7-20	GTGGGGATTTTCTGCTCAAA	TGGTTGGAAGGTACTGCAGA
In8-1	TTCTTCATTCTCACCATGCAA	GATTGCAAAGAGCCACAAAAC
In8-2	CCAATGTTGTGGTTGTGTAAACA	AAAATTGCACTCAACTGCACA
In8-3	AAACTTACAGTTCCGGCTGC	GTGCAATGAATTGATCATGGA
In8-4	GATCAATGGCCACGTGTACA	CCTAAGCCCACCAACTAGCA
In8-5	CTGAATGCTCTCTGGCTGTG	GTTTTGAACGAGCTTCTGCC
In8-6	TGGAGCCGAGCTAGAGAAAA	AATCTTGATGTCCAAACCGG
In8-7	AGGGCAGTCTTCCATTGATC	CACAATCAGGCATGTTTGGA
In8-8	TGATACACGCCCAATACCAA	GCGACTAGGTCATCCAAGGA
In8-9	GTTTGGAATGATGCCTACCG	CTTTATGGTGCCCACTTTGC
In8-10	TGATGACGACGGGAATTAGAG	TAACCACTTGTTTGCCCTCC
In8-11	CTTTAGGGATATCCGCCACA	TTGGGCATAGATCGTGTGAG
In8-12	CAAGATGGGGTGAATTGGAT	TGGAGTGTGTGAGGATACAATTG
In8-13	GTGGAAGGTGAAGCATCCAT	ACGACGCATAAGCTCCAAAG
In8-14	CACCGTGCTGTCATGATTTC	TTTTCTCGAATGGCATCAAA
In8-15	AGCGCCAAGATTGTTGCTAA	CCCCATTGTTCCTCTTTACG
In8-16	CGTGGTTTGGATTGCTTTTC	AGGCATGGCTCGTTATTGAC
In8-17	AGCATGTGGAAGTGCATTGT	GCTACTGCGCATGTTCAAAA
In8-18	TCCGGATTTAACCGTTTTTG	CGCTAGATTTTGGTAACGGG
In9-1	CGTGCCATTGAAGAAGAACA	ACACATTCCAACATATTTAGCCA
In9-2	GGAACGAAACGAGATGGTGT	TGATTACCCCCAAATTCCAG
In9-3	GGGACGTTCGATTCGTTCTA	TCTTTCCATGTCGCATTCAC
In9-4	TCTGGCTTAAAGGCACAGGT	GGGTGTTGTCAAGTTCTGCA
In9-5	TCTCCGAGGATAGTGATGGG	TGGCTTTCTCCGTGAAAACT
In9-6	GAATTCAAAATACGTGTGGCA	TTTGTTCACGGTTGTGGAAA
In9-7	CCTCATTGCACCACACTCAC	ATCAAGTAAGCCGTGATCGC
In9-8	TCCTCCTGCTGTAGCACTGA	CCACAAGTTCATATGATAAGCCA
In9-9	AGTCATCTTGCTGAAGCCAG	AGCTTATGCCTACATGTGAGGTC
In9-10	TGAAGCCGTGCAATCATCTA	TGCATTTGTGTTGATCTCGA

In9-11	AAGCACACAACATATGGCCA	GTGCAATTGGTGGGGATTAT
In9-12	CATGGCTGCGATATTTGAAA	ACAGTTGCATGCATTGTGGT
In9-13	GATGAAAAAGCAACAAAGGGA	TGACAGCAAAAAGGTGCAAA
In9-14	ATGAAACCCCTTGTTGGATG	TTCGTCCCAATGAAGGAAGT
In9-15	GGAAAATAAATCGGATGGGA	GGGGAGGAAATAGTTGCTTTT
In10-1	TTGCACCAAGATACAAATTTCG	TCTTTCTAGGGGACTTTAGCCA
In10-2	ACATCATACTGGGTAACTTCTTTCAA	CGTCAAAGACGCATGAATTT
In10-3	AAAACCCGGAAGAGATTCTAACA	TGCCTATGAAAGGAAACAAAA
In10-4	CGAGAACCTTCCTGCACTCT	TGTCATCCAGAATTGGGGTT
In10-5	CCATTATCCTACCATGCGCC	CACTTGCGTTCACGATCCT
In10-6	TGCAGACTATATGCACCATTCA	GAAAAGGAATGGCAAATTGG
In10-7	CCATCTTGCTTGATTTTCCC	TGCCCTTTACAAGTTTTTGAAA
In10-8	TGGTGGGAGAATAAATGGCT	CTTCCAATTAGTGGGACCCA
In10-9	AGTATTTGTGCGCGTGTGAA	GACATGAATAGACCATGTTCCC
In10-10	GGATTGCATTTCCATCACGT	GCCCACAGTTCATACCCAGT
In10-11	ACTTTACCATCATGCCAGGC	CCAAATTCGAGAAAACATTGC
In10-12	AAGTACTGGGGCCTTCCAAT	GCAAAGAGCCAATTTCTTGC
In11-1	TGGAAACTGGGGAACTTACG	AAAACATCAATTGTTCGGCC
In11-2	AATCAGGTACAGCAAAAGAAAAA	GTAACGTGGTCCAGGGAAAA
In11-3	TCTTTGCGATGAAGTGCATC	GCATGTTGGAAGGTCCTTTG
In11-4	CCACTACTCTCATGCCTGCA	ACAGCTAAATGAGATCTTTATGGAAAA
In11-5	ACCAAACCATGAATTGCCTC	CTCAACTTCACGCTTCTCCC
In11-6	CACCTAATTGGAGAACGTGGA	TGAAGCTCTCAGCGTCAGG
In11-7	TGGCAGCATCACCTGAATAA	GAGGATGATAATGGCCAGGA
In11-8	TGCGAAGAAATTTTTGGAGAA	CTGTGGTGATTTCTGGGGTT
In11-9	GGTTTACCTGATCCACCACG	AAGCTAAAACCGCAACCCTT
In11-10	TACTTTCGACCCGATTCACC	ATGGCATGTTGACCTGGATT
In11-11	CCACAGAATCAAGATTGGGC	TGAATAGAGGAGGGTGGCTG
In11-12	CGGTGATGTCTTAACCGGAT	GCGTGCTTTGATTCAGAAAA

SSR markers

**Primer**	**Forward sequence**	**Reverse sequence**
SES1-1	TCCCTCCACTTTCCATTAAACA	TGCTTGATTTGTGCATTTCCTT
SES1-2	ATTCAAACAATGGGTCTACGC	TCTCTCTAATTTCGATTGCTGA

SES1-3	TCCATCTCATCTCCATCACTGT	AGTTGAGGACCGAACTGAGA
SES1-4	CGTAAGAAGTCCTGCGAGGA	CGCTCAAGTCAAGACCAAGG
SES1-5	TCTGGCCAAATCCTTTTCACT	ACCCAATCCAACTTACATCACA
SES1-5	TCTGGCCAAATCCTTTTCACT	ACCCAATCCAACTTACATCACA
SES1-6	ACCATTCAACCGCATCACCT	AGTCGCACATTTGTTGACGA
QDT-20	TAGGGGTTCGTGTTCAGCAC	GGCAAGCCTAAGGTGGTTAT
QDT-21	CGTAGTGGATTCCATTCAAGGT	AGGAAGTATTTCATTGGCGTGA
SES-MT-40	GGGTGTTACAGCTTGTAATGCA	ACGCCATTGTTTGCAGTTTGA
**Primer for Vector construction**		
**Primer**	**Forward sequence**	**Reverse sequence**
DUF1005-<br>GFP	AAGTCCGGAGCTAGCTCTAGAATG<br>GATCCGTGCCCCTTCGT	GTCCTCGAGACGTCTCTAGACTGCCTTA<br>ACTCCTTCCGGAG
GFP-<br>DUF1005	TGGACGAGCTGTACAGATCTATGG<br>ATCCGTGCCCCTTCGT	GGGCGGCCGCTTTAAGATCTCTACTGCC<br>TTAACTCCTTCCG
