# Supplementary material for: Mapping and Functional Characterization of Stigma Exposed 1, a DUF1005 Gene Controlling Petal and Stigma Cells in Mungbean (Vigna radiata)
Source: Front Plant Sci. 2020 Nov 19;11:575922. doi: 10.3389/fpls.2020.575922 (PMC7710877; doi:10.3389/fpls.2020.575922)
Supplement: Supplementary file 4 [file Data_Sheet_2.PDF]

Table S2. Single nucleotide polymorphisms (SNPs) in the genome region containing *se1* gene. The SNPs are identified by compariosn of sequences among reference sequence, Sulv1 and its mutant *se1*

Chr	Position	Reference sequence	Sulv1*	*se1**	region	Gene	Type
11	286152	C	C	T	intergenic		-
11	286444	C	C	A	intronic	*Vradi11g00290*	-
11	286549	A	A	G	exonic	*Vradi11g00290*	synonymous SNP
11	286712	T	K	T	exonic	*Vradi11g00290*	nonsynonymous SNP
11	287286	T	T	G	intronic	*Vradi11g00290*	-
11	287562	T	Y	-	intronic	*Vradi11g00290*	-
11	287715	G	-	R	intronic	*Vradi11g00290*	-
11	287755	A	A	T	exonic	*Vradi11g00290*	synonymous SNP
11	287915	A	A	R	intronic	*Vradi11g00290*	-
11	287927	T	T	C	intronic	*Vradi11g00290*	-
11	287984	A	A	T	intronic	*Vradi11g00290*	-
11	287989	G	R	G	intronic	*Vradi11g00290*	-
11	288173	G	R	-	exonic	*Vradi11g00290*	synonymous SNP
11	288556	G	K	G	exonic	*Vradi11g00290*	synonymous SNP
11	289217	G	G	A	intronic	*Vradi11g00290*	-
11	289362	A	A	G	exonic	*Vradi11g00290*	synonymous SNP
11	290451	G	G	A	intergenic		-
11	290527	T	T	C	intergenic		-
11	290602	T	T	C	intergenic		-
11	290834	G	G	A	intergenic		-
11	290845	T	T	A	intergenic		-
11	290923	T	T	W	intergenic		-
11	290961	A	A	G	intergenic		-
11	291279	T	T	A	intergenic		-
11	291366	C	C	T	intergenic		-
11	291377	C	C	T	intergenic		-

11	293543	G	G	C	intronic	*Vradi11g00300*	-
11	296219	G	G	A	exonic	*Vradi11g00300*	synonymous SNP
11	296431	C	M	C	exonic	*Vradi11g00300*	nonsynonymous SNP
11	296468	C	Y	-	intronic	*Vradi11g00300*	-
11	300750	A	A	G	intergenic		-
11	300998	T	T	C	intergenic		-
11	301033	G	G	A	intergenic		-
11	301317	C	C	T	intergenic		-
11	301702	A	A	G	intergenic		-
11	301805	T	T	C	intergenic		-
11	302173	A	A	G	intergenic		-
11	306515	G	R	-	intergenic		-
11	306517	A	W	-	intergenic		-
11	306518	A	W	-	intergenic		-
11	306524	G	R	-	intergenic		-
11	307387	G	R	-	intergenic		-
11	307413	A	M	-	intergenic		-
11	309787	A	W	-	intergenic		-
11	309792	C	Y	-	intergenic		-
11	309795	C	Y	-	intergenic		-
11	309836	A	R	-	intergenic		-
11	309846	G	R	-	intergenic		-
11	309867	A	R	-	intergenic		-
11	309902	A	R	-	intergenic		-
11	309907	A	R	-	intergenic		-
11	309919	G	R	-	intergenic		-
11	309935	C	S	-	intergenic		-
11	309937	T	Y	-	intergenic		-
11	309940	G	R	-	intergenic		-
11	309963	A	R	-	intergenic		-

11	309975	G	R	-	intergenic		-
11	309991	G	R	-	intergenic		-
11	310000	C	Y	-	intergenic		-
11	310002	G	R	-	intergenic		-
11	310003	G	R	-	intergenic		-
11	310009	G	R	-	intergenic		-
11	310015	A	R	-	intergenic		-
11	310018	T	K	-	intergenic		-
11	310030	T	W	-	intergenic		-
11	310066	G	R	-	intergenic		-
11	310078	A	R	-	intergenic		-
11	310096	T	Y	-	intergenic		-
11	310453	A	R	-	intergenic		-
11	310483	G	R	R	intergenic		-
11	310486	T	Y	Y	intergenic		-
11	310581	G	G	S	intergenic		-
11	310943	T	W	-	intergenic		-
11	311397	G	R	-	intergenic		-
11	311437	A	R	G	intergenic		-
11	311471	C	Y	-	intergenic		-
11	311484	A	R	-	intergenic		-
11	311556	A	R	-	intergenic		-
11	311563	A	R	G	intergenic		-
11	311601	A	M	C	intergenic		-
11	311637	G	R	-	intergenic		-
11	311751	A	R	R	intergenic		-
11	311757	G	R	R	intergenic		-
11	311781	G	R	-	intergenic		-
11	311788	T	Y	-	intergenic		-
11	311808	T	Y	-	intergenic		-

11	312050	G	R	-	intergenic		-
11	312051	A	R	-	intergenic		-
11	312081	G	R	-	intergenic		-
11	312120	C	M	-	intergenic		-
11	312190	T	Y	-	intergenic		-
11	312201	A	M	-	intergenic		-
11	312204	G	R	-	intergenic		-
11	313158	G	G	T	intergenic		-
11	313171	G	G	A	intergenic		-
11	313183	A	A	T	intergenic		-
11	313224	T	T	A	intergenic		-
11	313272	C	Y	C	intergenic		-
11	313297	G	G	A	intergenic		-
11	313304	C	C	T	intergenic		-
11	313336	A	A	T	intergenic		-
11	313351	C	C	A	intergenic		-
11	313354	T	T	C	intergenic		-
11	313365	A	A	G	intergenic		-
11	313391	G	G	A	intergenic		-
11	313399	T	T	C	intergenic		-
11	313413	G	G	A	intergenic		-
11	313439	T	T	C	intergenic		-
11	313467	C	C	T	intergenic		-
11	313476	T	T	C	intergenic		-
11	313483	C	C	T	intergenic		-
11	313491	T	T	A	intergenic		-
11	313911	T	T	C	intergenic		-
11	313915	A	A	G	intergenic		-
11	313936	A	A	T	intergenic		-
11	314013	C	C	T	intergenic		-

11	314016	T	T	G	intergenic		-
11	314049	G	G	A	intergenic		-
11	314225	A	A	G	intergenic		-
11	314256	A	A	G	intergenic		-
11	314385	G	G	C	intergenic		-
11	314770	C	Y	-	intergenic		-
11	317004	G	-	R	intronic	*Vradi11g00310*	-
11	317662	T	Y	-	intergenic		-
11	319485	T	-	Y	intergenic		-
11	319700	G	-	R	intergenic		-
11	320148	C	Y	-	intergenic		-
11	320787	A	-	M	exonic	*Vradi11g00320*	synonymous SNP
11	321800	C	Y	C	intronic	*Vradi11g00320*	-
11	323144	G	G	K	exonic	*Vradi11g00320*	synonymous SNP
11	323311	G	G	R	intronic	*Vradi11g00320*	-
11	325201	A	A	R	intergenic		-
11	325984	G	R	-	intronic	*Vradi11g00330*	-
11	328779	T	K	-	intergenic		-
11	329468	A	R	-	intergenic		-
11	329998	A	R	-	intergenic		-
11	331437	C	-	Y	intergenic		-
11	347264	T	-	Y	intronic	*Vradi11g00370*	-
11	347715	C	M	C	intergenic		-
11	354427	T	T	Y	intronic	*Vradi11g00380*	-
11	357899	A	R	R	intronic	*Vradi11g00380*	-
11	357954	T	K	K	intronic	*Vradi11g00380*	-
11	359422	A	A	M	exonic	*Vradi11g00390*	nonsynonymous SNP
11	361469	C	Y	C	intergenic		-
11	363415	G	R	G	intronic	*Vradi11g00400*	-
11	363880	A	A	T	intronic	*Vradi11g00400*	-

11	365319	C	-	Y	intronic	*Vradi11g00400*	-
11	366153	T	T	Y	intronic	*Vradi11g00400*	-
11	369139	C	-	Y	intronic	*Vradi11g00400*	-
11	369628	A	-	R	exonic	*Vradi11g00400*	synonymous SNP
11	370294	A	R	A	intronic	*Vradi11g00400*	-
11	373613	C	M	C	intronic	*Vradi11g00400*	-
11	374292	C	-	Y	intronic	*Vradi11g00400*	-
11	378605	A	A	W	intronic	*Vradi11g00400*	-
11	379051	C	C	Y	intronic	*Vradi11g00400*	-
11	379105	T	Y	T	intronic	*Vradi11g00400*	-
11	396894	T	Y	T	intergenic		-
11	396916	C	C	M	intergenic		-
11	402195	T	T	C	exonic	*Vradi11g00400*	nonsynonymous SNP
11	404096	T	T	Y	intergenic		-
11	406014	G	R	-	intronic		-
11	408014	C	C	Y	intergenic		-
11	408064	C	C	M	intergenic		-
11	408941	T	T	C	intergenic		-
11	408942	C	C	T	intergenic		-
11	409561	G	G	R	exonic	*Vradi11g00430*	synonymous SNP
11	413851	G	R	G	intronic	*Vradi11g00430*	-
11	415062	T	T	Y	intronic	*Vradi11g00430*	-
11	415071	C	Y	C	intronic	*Vradi11g00430*	-
11	419147	C	M	C	exonic	*Vradi11g00430*	nonsynonymous SNP
11	425749	A	R	A	intergenic		-
11	427341	G	R	A	intergenic		-
11	427353	G	R	A	intergenic		-
11	427356	G	K	T	intergenic		-
11	427389	C	S	G	intergenic		-
11	427426	C	Y	C	intergenic		-

11	427577	A	W	W	intergenic		-
11	427585	A	W	W	intergenic		-
11	427590	T	W	W	intergenic		-
11	427595	C	Y	Y	intergenic		-
11	427635	T	Y	Y	intergenic		-
11	427670	A	W	W	intergenic		-
11	427687	C	M	M	intergenic		-
11	427693	T	Y	Y	intergenic		-
11	427716	A	-	W	intergenic		-
11	427732	T	-	W	intergenic		-
11	427750	A	-	W	intergenic		-
11	430632	G	R	G	intronic	*Vradi11g00460*	-
11	432474	G	R	G	intronic	*Vradi11g00460*	-
11	435229	T	Y	T	intergenic		-

*B = C/G/T, D = A/G/T, H = A/C/T, K = G/T, M = A/C, R = A or G, S = C/G, V = A/C/G, W = A/T, Y = C/T, N = A/C/G/T
