# Supplementary material for: Mapping and Functional Characterization of Stigma Exposed 1, a DUF1005 Gene Controlling Petal and Stigma Cells in Mungbean (Vigna radiata)
Source: Front Plant Sci. 2020 Nov 19;11:575922. doi: 10.3389/fpls.2020.575922 (PMC7710877; doi:10.3389/fpls.2020.575922)
Supplement: Supplementary file 5 [file Data_Sheet_3.PDF]

Table S3. Insertions and deletions (InDels) in the genome region containing *se1* gene. The InDels are identified by compariosn of sequences between Sulv1 and its mutant *se1*

**Chr**	**Position**	**Sulv1**	***se1***	**Type**	**region**	**Gene**
11	287709-287711	TAG	-	deletion	intronic	*Vradi11g00290*
11	289040	-	T	insertion	intronic	*Vradi11g00290*
11	289501	-	A	insertion	intronic	*Vradi11g00290*
11	289778-289779	CT	-	deletion	intronic	*Vradi11g00290*
11	290326	-	CA	insertion	intergenic	
11	291065	-	TTT	insertion	intergenic	
11	300086	C	-	deletion	intergenic	
11	300772-300776	AAAAA	-	deletion	intergenic	
11	300959-300962	AATA	-	deletion	intergenic	
11	301983-301985	TCC	-	deletion	intergenic	
11	302069	-	AAG	insertion	intergenic	
11	302834-302836	ATT	-	deletion	intergenic	
11	309863-309865	ATA	-	deletion	intergenic	
11	309883	A	-	deletion	intergenic	
11	313501	-	CGTG	insertion	intergenic	
11	313906	A	-	deletion	intergenic	
11	314323	-	GTAATTT	insertion	intergenic	
11	314364	T	-	deletion	intergenic	
11	314448	C	-	deletion	intergenic	
11	334041	-	A	insertion	intronic	*Vradi11g00350*
11	355656	-	AT	insertion	intronic	*Vradi11g00380*
11	396861	-	A	insertion	intergenic	
11	427371	-	C	insertion	intergenic	
11	427601	A	-	deletion	intergenic	
11	427612	-	T	insertion	intergenic	
